# Changes in Muscle Contractile Properties after Cold- or Warm-Water Immersion Using Tensiomyography: A Cross-Over Randomised Trial

**DOI:** 10.3390/s20113193

**Published:** 2020-06-04

**Authors:** Esther Mur Gimeno, Francesco Campa, Georgian Badicu, Jorge Castizo-Olier, Elisabet Palomera-Fanegas, Raquel Sebio-Garcia

**Affiliations:** 1 School of Health Sciences, TecnoCampus, Pompeu Fabra University, 08302 Mataró, Spain; emur@tecnocampus.cat (E.M.G.); jcastizo@tecnocampus.cat (J.C.-O.); 2 Grup de Recerca en Atenció a la Cronicitat i Innovació en Salut, TecnoCampus, Pompeu Fabra University, 08302 Mataró, Spain; 3 Department for Life Quality Studies, University of Bologna, 47921 Rimini, Italy; francesco.campa3@unibo.it; 4 Department of Physical Education and Special Motricity, University Transilvania of Brasov, 500068 Brasov, Romania; georgian.badicu@unitbv.ro; 5 Unitat de Recerca i Bioestadística, Consorci Sanitari del Maresme, 08304 Mataró, Spain; epalomera@csdm.cat; 6 Department of Rehabilitation, Hospital Clínic de Barcelona, 08036 Barcelona, Spain

**Keywords:** electrodiagnosis, hydrotherapy, muscle contraction, tensiomyography

## Abstract

Muscle contractile properties in clinical practice are often measured using either subjective scales or high-cost, inaccessible equipment. In this randomised cross-over study, we aimed to explore the use of tensiomyography (TMG) to assess changes in muscle contractile properties after cold- and warm-water immersion. The muscle contractile properties of the biceps femoris (BF) were assessed using TMG in 12 healthy active men (mean age 23 ± 3 years, Body Mass Index 22.9 ± 1.3 kg/m^2^) before and after a 20-min warm- or cold-water immersion over a period of 40 min. Muscle displacement (Dm) and contraction time (Tc) were registered as the main variables of the study. There was a significant condition by time interaction for Dm (*p* < 0.01). Post hoc analysis showed that, compared to the baseline, there was an increase in Dm 40 min after warm-water immersion (*p* < 0.01) and a decrease at 10 min after cold-water immersion (*p* < 0.01). No significant effect was found for Tc. Our results indicate that muscle contractile properties are affected by water temperature and time after the immersion; therefore, these factors should be taken into account when water-immersion is used as a recovery strategy.

## 1. Introduction

The search for the optimal physical condition and the monitoring of physiological parameters in athletes have always been topics of study for researchers, trainers and coaches [1,2]. Water immersion has been used in both sports and rehabilitation for several decades and its effects on several physiological parameters, including inflammatory and metabolic markers, blood flow and nerve transmission are well-documented in the literature [3,4,5,6,7,8]. The effects of water immersion appear as thermal, mechanical and chemical effects, either alone or as mixed effects [9]. Hydrostatic pressure is associated with several changes, including a decrease in muscle activation and muscle strength as well as a fluid shift from the intracellular to the intravascular space, which may assist the process of eliminating metabolic wasting [4,7]. The application of heat is typically explained by its effects on vasodilation and increase in blood flow and metabolism, which should cause healing to occur more quickly [9]. On the other hand, cold is frequently used in sports rehabilitation because of the reduction of pain, swelling and inflammation associated with it [4,7]. Although these physiological changes are well-known, the effects of water temperature on muscle mechanical properties has been studied less. 

Muscle contractile properties (velocity of contraction, time to maximal contraction, fiber composition, etc.) are a key determinant of strength and power [10]. Several tools have been developed depending on the assessed properties, such as torque recordings, mechanomyography, magnetic resonance, muscle biopsy and performance variables, such as the countermovement jump test (CMJ) [10,11]. In recent years, tensiomyography (TMG) has been proposed as an alternative method to assess muscle contractile properties, including muscle stiffness, contraction speed, muscle composition and muscle fatigue in different muscles (biceps femoris, vastus lateralis, rectus femoris, among others) [12,13,14,15,16]. This method is based on the assumption that the radial muscle belly displacement detected by a digital sensor represents the enlargement of the muscle surface due to contraction, which will be proportional to muscle force [13,16]. Although TMG does not measure muscle strength directly, some studies have shown a good correlation with the estimated one maximum repetition (1RM) and maximal voluntary isometric contraction [10,17]. On the other hand, as contraction time depends on the type of fiber composition, this parameter has been effectively used to assess muscle composition [13]. The advantages of TMG over other methods to assess muscle contractile properties include the ability to isolate the action of a single muscle group and the fact that it doesn’t rely on voluntary muscle contraction [12,18]. These features are especially relevant for rehabilitation purposes as well as to objectively discern between effective muscle contraction and potential psychological interferences, such as readiness or fatigue, that might influence muscle performance. In terms of reliability, TMG has shown excellent inter-rater reliability (intra-class correlation coefficient ICC ranging from 0.77–0.97) [19,20] as well as between day repeatability (ICC = 0.94) [21] and reproducibility at different sensor positions [22]. According to this, it has been recommended as a consistent method to assess muscle contractile properties [18]. 

To date, very few studies have analysed how water immersion may affect the contractile properties of the striated muscle using TMG [22], with no studies exploring the effects of different water temperatures. Therefore, the aim of this study was two-fold: (1) to examine the effects of warm-water immersion (WWI) and cold-water immersion (CWI) on the biceps femoris’ (BF) contractile properties using TMG and (2) to explore the interaction between water temperature and time post-exposure. Given the aforementioned effects of water temperature on nerve transmission, we hypothesized that WWI would elicit an increase in radial muscle displacement, while the opposite would be observed after CWI, especially during the first minutes after exposure. 

## 2. Materials and Methods

### 2.1. Design

We conducted a cross-over single-arm pilot study at a Laboratory for Athlete High Performance over a period of two months (October and November). 

### 2.2. Subjects

Fifteen healthy men were screened for inclusion. To participate in the study, the only inclusion criteria were to engage at least in 10 h of exercise training per week. Participants were excluded (A) if they presented any significant neuromuscular, musculoskeletal, traumatic injury or any other impairment in the assessed limb over the previous six months (such as fibrillary rupture, ligament sprain, fracture, dislocation or meniscus injury) and (B) if they reported extenuating exercise or consumption of caffeinated-drinks in the 48 h previous to any of the assessments. Three subjects were excluded because one had suffered an ankle sprain during the previous 6 months and two had consumed some sort of energy-drink in the 48-h previous to the first evaluation. Thus, twelve men participated in the study. 

The sample size was calculated based on a previous study published by García-Manso et al. [23] where the authors reported a decrease of 1.75 mm in maximal muscle displacement after cold-water immersion. With a statistical power of 80% and a confidence interval of 95%, assuming a standard deviation of 2.27 mm, we needed to recruit at least 10 subjects. 

The project was conducted in accordance with the guidelines of the declaration of Helsinki, and was approved by the local Bioethics Committee (Approval code: 25027). All subjects were properly informed about the study’s purpose, and written consent was obtained before any formal testing. 

### 2.3. Procedures

**Figure 1:**
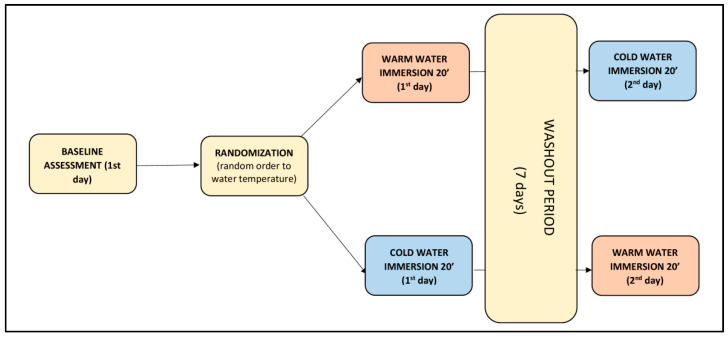
Measurement protocol on two non-consecutive days (washout period 7 days).

The study was conducted on two different days separated by one week. Each day, participants underwent the same evaluations. First, anthropometric measurements were taken according to the International Society for Advanced of Kinanthropometry (ISAK) [24]. A baseline TMG measurement was performed followed by a 20-min cold- or warm-water immersion. Post-immersion TMG measurements were taken immediately post-immersion and were repeated subsequently at 10, 20, 30 and 40 min thereafter. Allocation to CWI or WWI was performed randomly using a random generator program (Epidat 4.2 Xunta de Galicia) and results were placed in opaque envelopes by a third person not directly involved in the study. Between measurements, participants rested on a bench covered with a bathrobe and sweatshirt (Figure 1). 

### 2.4. Measuring Protocol

**Figure 2:**
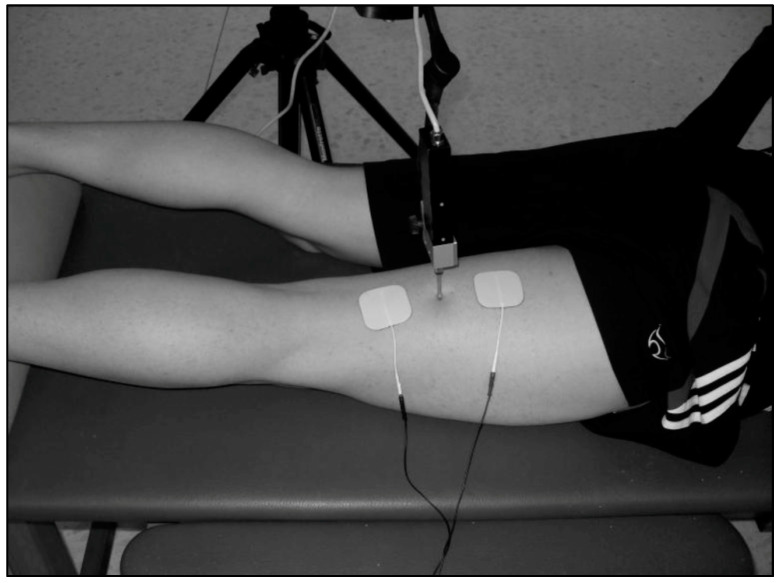
Position of the dominant knee and sensor tip perpendicular to the muscle belly, with electrodes symmetrically placed 3 cm from the sensor.

TMG measurements were taken in a laboratory at 22 ± 1 °C temperature. Measurements were registered with participants in a prone position with knee flexed at 20° (0° corresponding to full extension). The measured limb was positioned with the ankle joint placed on a triangular foam pad to keep a fixed knee angle. Rotation of the hip was maintained in a neutral position and the joint was left loose to avoid an isometric contraction (Figure 2). A digital displacement transducer (GK 40, Panoptik d.o.o., Ljubljana, Slovenia) which incorporates a spring of 0.17 N mm^−1^, providing an initial pressure of 1.5 × 10^−2^ N/mm^2^, was set perpendicular to the muscle belly to obtain BF radial displacement [25]. Sensor location was determined anatomically according to the references provided by Delagi et al. [26]. The investigator asked the participant to bend the knee from the resting position while applying resistance to locate the muscle belly borders, and subsequently marked the midpoint of the imaginary line between the fibula head and the ischial tuberosity with a dermatological pen [25]. Two square-shaped (4.5 × 4.5 cm) 2 mm-thick self-adhesive electrodes (RM 4545 Rehab Medic^®^, Barcelona, Spain) were placed symmetrically distal and proximal to the sensor tip (3 cm each way) and a TMG-S1 stimulator (EMF-Furlan I Co. d.o.o., Ljubljana, Slovenia) was used. Electrical stimulation was provided by means of a single square pulse of 1 ms, and intensity was initially set at 50 mA and increased 10 mA every 10−15 s until no further change in Dm was observed or maximal stimulator output was achieved (110 mA) [19,27]. The positive electrode was placed proximally while the negative electrode was placed distally. Baseline parameters were obtained after two consecutive measurement protocols separated by 5 min, and mean values were used for analysis. 

### 2.5. Water Immersion Protocol

Water immersion was performed with participants seated in a Jamaica massage bath tub of 250 L capacity, with their back supported on the back rest, arms relaxed outside the tub and legs placed semi-flexed on a weighted physiotherapy wedge and water level up to the fourth lumbar vertebra. Participants were asked not to eat or drink during the 20-min immersion. Water temperature was monitored continuously using a water thermometer to maintain a 10 ± 1 °C and 42 ± 2 °C during cold- and warm-water immersion, respectively, while room temperature was kept at 22 ± 1 °C. 

### 2.6. Outcomes

The main outcomes of the study were changes in TMG parameters (Dm and Tc) pre- to post-immersion according to water temperature. TMG parameters were obtained during BF contraction from the displacement-time curve, as described by Valenčič et al. 1997 [12]. Maximal displacement in millimitres (Dm) and contraction time in seconds (Tc) measured as the time from 10% to 90% of the maximal displacement curve, are the easiest parameters to reproduce (intra-class correlation coefficient 0.82–0.99 and 0.70–0.99, respectively) and more reliable [19,20,22]; hence we only selected those for analysis purposes. Since Dm is believed to represent the contraction of the muscle, lower scores are indicative of a high degree of muscle tone or muscle stiffness [14,17]. Similarly, contraction time (Tc) has been found to be strongly correlated to muscle type distribution, with lower Tc values being found in fast-contracting muscles [25]. 

### 2.7. Statistical Analysis

A descriptive analysis of the main categorical and continuous variables was performed initially, prior to further analysis. Continuous variables are expressed in mean and standard deviation (SD) while categorical variables are represented in absolute values and corresponding percentages. Distribution was assessed for every continuous variable using the Shapiro–Wilk test. Log10 transformation was performed when variables were not normally distributed. A repeated-measures analysis of variance (ANOVA) was performed with time (PRE, POST, POST10′, POST20′, POST30′, and POST40′) as within-subject variables, and temperature (cold water and warm water) as the between-subject factor. When a significant F ratio was obtained, a Bonferroni post hoc test was used to evaluate time and temperature differences. Partial eta-squared effect sizes were also calculated to account for the effect of time and temperature on both Dm and Tc. Statistical analysis was performed using the statistical software SPSS v.21, IBM^®^ (IBM Corporation, Chicago, IL, USA), with a statistically significant level set at <0.01 according to the Bonferroni correction for multiple comparisons. 

## 3. Results

**Figure 3:**
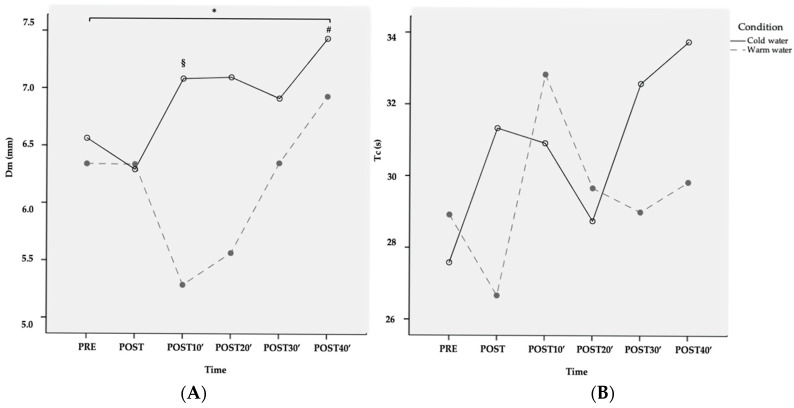
Changes in Dm (**A**) and Tc (**B**) after CWI and WWI (raw data—variables non-transformed). * = Significant interaction condition by time; § = Differences of cold-water condition with respect to PRE; # = Differences of warm-water condition with respect to PRE.

Twelve men (mean age 23 ± 3 years, BMI 22.9 ± 1.3, minimum leg perimeter 51.7 ± 3 and maximum leg perimeter 56.2 ± 2.8 cm) completed the study and were included in the statistical analysis. Results from the ANOVA are shown in Table 1. There was a significant condition by time interaction (*p* < 0.01) for Dm. Pairwise comparisons showed that, compared to the baseline, CWI resulted in a decrease in Dm 10 min post-exposure (mean change −0.09 ± 0.08; 95% CI: −0.14, −0.03; *p* = 0.005, transformed variable) while after warm-water immersion, an increase in Dm was observed at 40 min (mean difference +0.06 ± 0.05 95% CI: 0.02–0.09, *p* = 0.003, transformed variable). Significant differences between water temperatures were found 10 min post-immersion (mean difference = 0.13 ± 0.12 95% CI: 0.06–0.21, *p* = 0.003). No other differences in Dm were found between water temperatures (Figure 3A). No significant interaction and time effects were found for Tc (Figure 3B). 

## 4. Discussion

This study aimed to explore the biceps femoris’ muscle contractile properties over time using TMG after either warm or cold-water immersion to assess potential differences between water temperatures. This study found a significant interaction between water temperature and time for muscle displacement. Although no definitive conclusion can be drawn given the lack of intra-muscular temperature assessment, this might indicate that water temperature could change muscle contractile properties over time. Particularly, WWI for 20 min resulted in an increase in muscle displacement at 40 min compared to the baseline, suggesting a decrease in muscle stiffness and muscle tone [19]. This finding was not observed after cold-water immersion. Furthermore, the difference found between water temperatures at 10 min points to a short-term increase in muscle stiffness shortly after the application of CWI. This information might assist sports related professionals in determining when and how to adopt water immersion as a recovery strategy.

To the best of our knowledge, this study is the first to analyse the effects of both warm- and cold-water immersion on the muscle contractile properties of the BF using TMG. Previously, García-Manso et al. [23] examined the effects of four short (four minute) intermittent immersions in cold water on the muscle contractile properties of the vastus lateralis after each immersion. Particularly, they observed a reduction in the Dm of up to 34% over time, which was statistically significant for the last two immersions compared to the baseline. Similarly, in our study, cold-water immersion significantly decreased muscle displacement post-application. The decrease observed in Dm both in Garcia-Manso’s study and our study with cold-water is consistent with most of the literature, and is probably caused by the reduction in intra-muscle temperature observed with the application of cryotherapy in relation to the range of normal function temperature of the body (±36–37 °C). Studies have shown that a 7–8 °C degree reduction in intramuscular temperature occurs after 15–20 min of cryotherapy [28,29]. The measurement of intra-muscular temperature is an invasive procedure which requires the presence of trained personnel and sophisticated equipment, which is not functional for daily practice. Currently, no single parameter is able to adequately predict the change in intra-muscular temperature. Measurement of skin temperature has been proposed as an indirect assessment of muscle temperature but, according to some studies, skin temperature explains very little of the variance observed in intramuscular temperature [30]. However, unlike the study conducted by García-Manso et al. [23], where Dm was found to decrease further with each subsequent immersion, in our study the only difference pre-immersion was found at 10 min. The differences found between both experiments could be explained by the temperature used (4 °C vs. 10 °C) but also by the duration of the immersion (short intermittent immersions versus a continuous 20-min immersion). We decided to choose 10 °C and 42 °C respectively for 20-min based on the available literature which suggested temperatures ranging between 10–15 °C for CWI and >36 °C for WWI with applications lasting at least 10 min to yield results [4,6,8,31]. However, a systematic review published shortly afterward concluded that 11–15 min of CWI is considered to provide the best results; therefore, future research should take this timeframe as optimal [31]. However, 20 min of continuous application is in line with another recent study [32] and it shouldn’t lead to a very different response than the 15 min suggested by Machado et al. [33].

In this study, warm-water immersion was followed by an increase in Dm 40 min post-exposure (mean change +0.06 ± 0.05, 95% CI: 0.02–0.09). We believe this increase in Dm after warm-water immersion is caused by the effects derived from the application of heat on the autonomous nerve system and, particularly, on the relaxation that occurs as a consequence of the activation of the parasympathetic system [7]. Given that Dm measures the radial displacement of the muscle belly, it has been regarded as an indirect measure of muscle stiffness and muscle tone [13,14,15,34]. Based on this assumption, our results suggest that warm-water immersion could be more effective than cold water to decrease muscle tone and muscle stiffness, especially shortly after application. Our findings are in line with recent studies, such as the study conducted by Point et al. [28] where they reported an increase in muscle stiffness measured with ultrasonography and a decrease in muscle temperature after 20 min of air-pulsed cryotherapy. In accordance to what we observed in our study, no differences were found compared to the baseline for 40 min after application, suggesting that the effects tend to disappear within time.

Contraction time is the other TMG-parameter most frequently analysed in the literature. In our study, we observed substantial variability in Tc with no apparent effect of time or temperature, nor the interaction of both factors. This finding is consistent with the study published by Garcia-Manso et al. [23] where the authors reported a non-significant increase between 1.8% and 9% after the first, third and fourth immersions. As explained in the introduction section, Tc reflects the time elapsed between 10% and 90% of maximal muscle displacement [12] and it is mostly influenced by muscle composition [25]. According to the literature, an increase in Tc relates to a reduction in contraction velocity, leading to a compromise in muscle power. This assumption is currently supported by the literature, suggesting that the application of cryotherapy might hinder muscle performance within 40 min after application [28]. Unfortunately, in our study we failed to demonstrate a significant change in Tc after either cold- or warm-water immersion. This finding could be explained considering that our study was powered to find differences in muscle displacement and not contraction time, and because of the strong correlation between contraction time and fiber composition. Future studies should focus on other measurements of muscle reaction time, such as delay time or contraction velocity, instead of Tc.

Although TMG has shown good inter-rater reliability as well as excellent repeatability and reproducibility to assess muscle contractile properties in different contexts, there are some concerns that should be taken into consideration when it comes to a generalization of the results. For example, time inter-stimulus (referring to the time elapsed between two consecutive stimuli) can affect the results obtained with TMG. An interval of 10 s has been suggested, as it results in higher Dm values [27]. Inter-electrode distance is another crucial factor for TMG measurements. Tous-Fajardo et al. [19] suggested in their protocol that an inter-electrode distance of 5 cm increased maximal Dm compared to 3 cm. More recently, a study conducted by Wilson et al. [27] showed that increasing the distance 2 cm more (from 5 cm to 7 cm) further increased maximal Dm. In our study, we applied the same protocol previously published by Tous-Fajardo et al. [19] in 2010, given their positive results in terms of reproducibility and reliability. However, in light of this new evidence, future studies should be performed to compare different inter-electrode distances, possibly in relation to the length of the muscle, in order to work towards a standardized protocol.

This study has some limitations that should be addressed. First of all, this is an observational study with no control group; therefore, we can’t isolate the effects of water temperature on Dm from those caused by hydrostatic pressure. However, the effects of hydrostatic pressure on blood flow are usually measured with the subject in vertical position which will result in a larger effect given that hydrostatic pressure increases in relation to the depth of immersion [4]. Therefore, it is unlikely that the effects of hydrostatic pressure in the position adopted in this study are responsible for the effects found in Dm, and they would certainly not explain the differences observed between temperatures. Another important issue is that subjects were only men; therefore, the results cannot be translated into a female population, which may show a different pattern/behavior, given the differences in body and muscle composition [34]. More importantly, as mentioned in the discussion, we did not measure the intra-muscular temperature, which is likely to influence the results of the study. Measuring intra-muscular temperature is an invasive procedure which is unlikely to happen in non-experimental situations. Although considerably accurate predicted models of intra-muscular temperature have been developed in the literature, according to the authors, such predictions are probably not practical during most clinical treatments; thus, the applicability is poor.

## 5. Conclusions

In summary, our results indicate that WWI for 20 min results in a significant increase in muscle displacement 40 min post-exposure which, according to the literature, suggests a decrease in muscle stiffness. In addition, CWI decreased muscle displacement 10 min post immersion, suggesting a short-term increase in muscle stiffness. These findings should be taken into consideration when applying CWI as a recovery strategy, especially if exercise is going to resume shortly thereafter. Future studies involving both men and women are needed as well as studies considering immersion in thermoneutral water, contrast therapy or kneipping as control group to corroborate our findings and to make assumptions for field and clinical practice.

## Figures and Tables

**Table 1:** Two-way ANOVA for the comparison of muscle displacement and contraction time changes over time, according to water temperature.

		PRE	POST	POST10′	POST20′	POST30′	POST40′	ANOVA		
		Mean ± SD	Mean ± SD	Mean ± SD	Mean ± SD	Mean ± SD	Mean ± SD	Time Effect	Time × Temperature	Temperature Effect
Log10(Dm)	C	0.75 ± 0.11	0.75 ± 0.11	0.66 ± 0.12 *	0.67 ± 0.07	0.75 ± 0.10	0.79 ± 0.11	F = 6.6; *p* < 0.001; η^2^*_p_ =* 0.23 *	F = 6.1; *p* < 0.001; η^2^*_p_ =* 0.21 *	F = 18.1; *p* < 0.001; η^2^*_p_ =* 0.21 *
	W	0.77 ± 0.10	0.74 ± 0.10	0.79 ± 0.15 ^#^	0.79 ± 0.13	0.79 ± 0.15	0.82 ± 0.11 *			
Log10(Tc)	C	1.43 ± 0.09	1.48 ± 0.09	1.47 ± 0.14	1.45 ± 0.07	1.49 ± 0.10	1.51 ± 0.12	F = 1.4; *p* = 0.240; η^2^*_p_ =* 0.05	F = 1.5; *p* = 0.304 η^2^*_p_ =* 0.06	F = 1.9; *p* = 0.162; η^2^*_p_ =* 0.03
	W	1.45 ± 0.09	1.41 ± 0.09	1.49 ± 0.14	1.44 ± 0.15	1.44 ± 0.11	1.46 ± 0.09			

Note: W = Warm water; C = Cold water; * = *p <* 0.01 vs. PRE; ^#^ = *p* < 0.01 between temperatures. Variables are presented after log10 transformation.
